# Pharmacological Mechanism of Pingxiao Formula against Colorectal Cancer

**DOI:** 10.1155/2022/7884740

**Published:** 2022-12-20

**Authors:** Wei Yu, Chang Lu, Guoliang Wang, Zhenghao Liang, Zizheng Jiang, Yanzhi Liu, Jing Yan

**Affiliations:** Department of Physiology, Jining Medical University, Jining City, Shandong Province, China

## Abstract

Colorectal cancer (CRC) is the most common cancer worldwide and develops due to a broad range of causative factors. Pingxiao (PX) formula and Xihuang (XH) formula are two commonly used drugs to treat CRC, especially as an alternative therapy for those patients who could not suffer surgery, chemotherapy, or immunotherapy, namely, elder or advanced CRC patients. However, the pertinent pharmacological mechanisms are still elusive. The investigation was designed to explain the pharmacological mechanisms of the PX formula. A murine model of CRC was established by injecting CT26.WT cells into the caecum of 4-week-old male Balb/c mice, following PX or XH treatment for 30 days. Network pharmacology analysis combined with weighted gene coexpression network analysis (WGCNA) predicted the pharmacological mechanisms and therapeutic value. High-throughput 16S rRNA sequencing determined the alterations in the gut microbiota communities. Western blotting, immunofluorescence, and flow cytometry examined the influence of PX on the tumor microenvironment (TME). Injection of CT26.WT-induced CRC in Balb/c mice was markedly attenuated by PX treatment. Compared with XH administration, PX exhibited a stronger antitumor effect, such as smaller tumor volume, lower interleukin 17 (IL-17), IL-6 and tumor necrosis factor-alpha (TNF*α*) serum levels, and higher interferon-gamma (IFN-*γ*) concentration. Network pharmacology analysis demonstrated that both PX and XH targets were enriched in cancers and inflammatory responses. RNA sequencing confirmed that PX treatment induced cancer cell apoptosis and inhibited inflammatory reactions within the tumor. Moreover, the PX formula considerably restored homeostasis of the gut microbiota, which was not observed in the XH group. PX targets, those associated with the survival probability of CRC patients, correlated with macrophage (M*φ*) infiltration, which presented an independent risk factor for the CRC outcome. PX treatment promoted the transition of alternatively activated M*φ*s (M2 M*φ*s) to classically activated M*φ*s (M1 M*φ*s). Moreover, the peritoneal M*φ*s from the PX group inhibited the migration of CW26.WT cells, as evidenced by the wound healing experiment and transwell assay, which was consistent with the decreased expression of the vascular endothelial growth factor (VEGF). Furthermore, the coculturing system confirmed that PX-treated M*φ*s suppressed colorectal tumor-derived organoid proliferation. PX formula exhibits a potential antitumor effect against CRC by suppressing the colonization of pathological microorganisms, reshaping M*φ* effector functions and hence inhibiting cancer cell proliferation.

## 1. Introduction

Colorectal cancer (CRC) is one of the most common cancers and the third most frequent cause of cancer-related deaths, and CRC-caused mortality has increased by 40% with the development of economics during the decades. Despite the access to specialists and effective therapy in recent years, it has been projected that there has been a rise in the number of young patients as well as subsequent deaths [1–3]. The pathogenesis of CRC involves the environment, genetic susceptibility, nutrition, gut microbiota, and the natural history of inflammatory bowel disease [4]. Surgical resection is the only curative choice for patients with CRC, followed by adjuvant chemotherapy or immunotherapy, especially for those who have clinically occult micrometastasis. However, for patients who are not suitable for surgery or long-period chemotherapy, namely, elderly or terminal cancer patients, adjuvant drug therapy is the backbone of anticancer treatment [5]. In China, traditional Chinese medicine (TCM) is widely administered after surgical resection to avoid tumor recurrence and metastasis due to its lower cost and lower toxicity than other adjuvant options. Especially for elderly patients with advanced CRC, TCM has been considered the only palliative therapy. More importantly, given that the pathogenesis of CRC, a TCM formula, mostly composed of several ingredients that are anticancer, immunity-promoting, and anti-inflammatory, is promising to modulate the tumor microenvironment (TME), which determines tumor progression and outcomes. Multiple traditional Chinese anticancer drugs are utilized in the clinic to combat various cancer types including Pingxiao (PX) formula, Xihuang (XH) formula, and Xiangshaliujunzi decoction, stated Su et al. [6].

The PX formula is an anticancer drug and encompasses alunite, *Toxicodendron vernicifluum* (Stokes) F. A. Barkley (TV), *Strychnos nux-vomica* L. (SS), nitre, *Agrimonia pilosa* Ledeb (AL); *Curcuma aromatica* Salisb. (CR), *Citrus* × *aurantium* L. (CL), *Feces trogopterus* (FT) (Table 1). The PX formula is an improved formula to protect against blood stasis, tubercles, and tumors by a Chinese physician, Jia Kun on the basis of “Xiaoshi-Fanshi-San” in Jin Gui Yao Lue (written by Zhang Zhong Jing, a Chinese pharmacologist of the Eastern Han dynasty) composed of alunite and nitre to treat liver and gall disorders. Its chemical compositions and metabolites in rats have been identified by untargeted data-dependent tandem mass spectrometry [7], and its efficacy against liver cancer and CRC has been investigated in the clinic [8, 9]. However, these studies are limited in subjects and unknown pharmacological mechanisms. There is an unmet need for more detailed experimental evidence of its efficacy in treating CRC. In this study, we aimed to delineate the molecular and cellular mechanisms of the PX formula against CRC by assessing the anticancer effect, predicting the therapeutic value in the clinic, and demonstrating its influence on TME.

## 2. Materials and Methods

### 2.1. Network Analysis

All the plant names have been checked with https://www.theplantlist.org. Component-target networks were computed by the active components according to the following criteria: 0.18 drug-likeness and 30% oral bioavailability. The associated genes of CRC were collected from five databases: GeneCards, Online Mendelian Inheritance in Man (OMIM), DrugBank, PharmGkb, and Statistics of Therapeutic Target Database (TTD). The targets of active components were obtained from PubChem and the Traditional Chinese Medicine Systems Pharmacology Database and Analysis Platform [10]. The ingredient-target interaction network was produced by Cytoscape software. The data of the protein-protein interaction (PPI) network were obtained from the Search Tool for Retrieval of Interacting Genes/Proteins database with a confidence score ≥0.7 and calculated by CytoNCA [11], sorting the data based on the median values of betweenness centrality (BC), closeness centrality (CC), degree centrality (DC), local average connectivity (LAC), eigenvector centrality (EC), and network centrality (NC).

### 2.2. Clinical Patient Data Acquisition and Consensus Clustering Analysis

The mRNA expression profile and clinical information of 426 patients with colon adenocarcinoma (COAD) were downloaded from The Cancer Genome Atlas (TCGA) database, and weighted gene coexpression network analysis (WGCNA) was performed. The tumor samples were clustered into two groups based on the mRNA levels of the PX targets by the “limma” and “ConsensusClusterPlus” packages. The Kaplan–Meier (KM) plotter was plotted to utilize “survival” and “survminer” [12] packages. In the least absolute shrinkage and selection operator (LASSO) regression was computed by package “glmnet” to predict which PX-targets were associated with the survival probability with the penalty regularization parameter (*λ*). Based on the optimal lambda value as well as the corresponding coefficients, the risk signature genes were obtained and samples were clustered into low-risk and high-risk groups. The receiving operating characteristic (ROC) curve with the area under the curve (AUC) representing the degree or measure of separability was constructed by “pROC” and “ggplot2” packages to determine the discrimination ability. The closer to 1 means the validity. Single-sample gene set enrichment analysis (ssGSEA) was performed to identify the influenced immune cells by PX treatment.

### 2.3. Murine Model of CRC and Treatment

Experiments were conducted under the supervision of the guidelines of the Institutional Animal Care and Use Committee of Jining Medical University in China (SYXK-Shandong province 2018-0002).

In the PX formula, the weight ratio of CL, SS, nitre, AL, CR, alunite, FT, and TV is 5 : 3:3 : 3:3 : 2.5 : 1. Mixed CL, nitre, CR, alunite, FT, and TV powder were extracted by 70% ethanol, and the leftover and AL were boiled and concentrated. AL was added to the dried mixture. A total of 405 g of powder was obtained about 230 g of PX drug. To validate the components, a connected system of the LC-30 (Shimadzu)-hybrid quadruple time-of-flight mass spectrometer (TOF-MS) with an electrospray ionization source (ESI) was used. The mobile phase system had solution A (acetonitrile) and equate B (0.1% HCOOH-H_2_O): 25 min (A:20%:B:80%), 50 min (A:35%:B:65%), 9 min (A:90%:B:10%), and 7 min (A:20%:B:90%). Data were processed in information-dependent acquisition (IDA) with a high sensitivity mode. XH Wan was bought from the Beijing Tong Ren Tang drug company.

10^5^ CT26.WT cells were injected into the caecum of male Balb/c mice (Pengyue Animal Center of Shandong province, China) (male, about 22 g, 2 months old), and on the same day, the mice were treated with PX or XH by oral for the next 30 days. The dose for a human being is 300 mg/kg per day based on the Chinese Pharmacopoeia 2020, and the correspondingly murine is 3.6 g/kg calculated by the Meeh Rubner formula: skin surface area = mass coefficient^*∗*^weight^*∗*^2/3^*∗*^10^−4^. The positive control of the study is cyclophosphamide (CTX) (MedChemExpress, China) which facilitates antitumor immunity and suppresses CRC progression [13]. 90 mg (equal to 3.6 g/kg) or 45 mg (equal to 1.8 g/kg) were diluted in PBS and orally given to mice each day. Every 15 mice were grouped, and six groups were set as follows: 0.9% saline group, low concentration of the PX-treated group (1.8 g/kg), high concentration of the PX-treated group (3.6 g/kg), low concentration of the XH-treated group (1.8 g/kg), high concentration of the XH-treated group (3.6 g/kg), and the CTX (200 mg/kg) group. On day 31, isoflurane (RWD Life Science, Shenzhen city, China) was used for anesthetization. Tumors were collected and measured.

### 2.4. Microbiota and Microbiome Analysis

The feces and mucus within the colon were collected and mixed and processed by 16S rRNA sequencing. Raw data were filtered [14] and produced paired-end reads. Corrected paired-end reads generated circular consensus sequencing (CCS) reads. After removing chimeras, OTU (operational taxonomic unit) analysis was performed with a similarity >97%. Krona, as a powerful metagenomic visualization tool, demonstrates species annotation. Each fan means the corresponding annotation's proportion [15]. PICRUSt (Phylogenetic Investigation of Communities by Reconstruction of Unobserved States) analysis was conducted to predict the alterations in KEGG pathways. The raw data are available with the accession number in SRA (PRJNA820603, PRJNA796207).

The feces and mucus sample was treated with methanol and standard internal substances. After the ultrasound and frozen treatment, the samples were centrifugated. The supernatant was subjected to ultrahigh-performance liquid chromatography (UHPLC) coupled with TOF-MS (Garcia and Barbas, 2011). Acquisition software (Analyst TF 1.7, AB Sciex) was utilized to assess the full scan survey MS data, which were then converted by ProteoWizard and generated the retention time (RT), mass-to-charge ratio (*m*/*z*) values, and peak intensity (Salido et al., 2009). According to the In-house MS2 database, substances were identified and determined by orthogonal projections to latent structures-discriminant analysis (OPLS-DA). The cut-off is fold change (FC) > 1, *P* value <0.05, and variable importance in the projection (VIP) > 1 [16].

### 2.5. RNA Sequencing

FastQ files were processed for primary quality control. Read pairs that contain more than 3 or the proportion of bases with a quality value below 5 were removed. All the downstream analyses were completed on the clean data obtained. Differentially expressed genes (DEGs) were identified using the DEGSeq R package (PRJNA820958).

### 2.6. PX Serum Isolation

After 3.6 g/kg PX treatment, we collected the serum from CRC mice in two hours. To examine the toxicity, CT26.WT cells were treated with the serum and subjected to an MTT assay (3- [4,5-dimethylthiazol-2-yl]-2,5-diphenyl tetrazolium bromide) (Supplementary Figure 3).

### 2.7. Macrophage (M*φ*) Culturing and Phagocytosis Experiment

For peritoneal M*φ* preparation, mice were anesthetized and sterilized PBS was injected into the abdominal cavity. After the massage, the liquids were isolated and centrifuged. The formula was diluted in a 1640 complete medium.

For the bone marrow-derived macrophage (BMDM), 4 week-old male mice were sacrificed and bone marrow from femurs was incubated with blood cell lysis buffer (Beyotime, China) for 5 minutes. Filtered cells were cultured with the 1640 medium in the presence of the granulocyte-macrophagecolony-stimulating factor (STEMCELL Technologies, Canada) at a concentration of 55 ng/ml. M*φ*s were collected on day 7.

10^5^ M*φ*s were cultured with prepared microparticle solution (Thermo Fisher, USA) for 2 hours at 37°C and then mounted at the fluorescein isothiocyanate (FITC) wavelength (488 nm).

### 2.8. Colorectal Tumor Organoid Culture and Coculturing

The solid tumor was cut into small pieces and washed with cold PBS until the PBS solution became transparent and clear. After 35 minutes of digestion by digestive solution [17], the filtered medium was centrifugated and cultured in a growth medium. After 9 days, colorectal tumor-derived organoids (cTDOs) were cocultured with PX-treated peritoneal M*φ*s. After 24 hours, cTDOs were incubated at 37°C with propidium iodide (PI)/Hoechst33342 (480 nm/630 nm) for 20 minutes. For Ki67 staining, the Ki67 eFluor™ 660 eBioscience™ (Thermo Fisher, USA) (1 : 1000) antibody was incubated for 24 hours and examined by using a fluorescent microscope (Zeiss, Japan).

### 2.9. Western Blotting

The total protein (40 *μ*g) was subjected to SDS-PAGE (sodium dodecyl sulfate-polyacrylamide gel electrophoresis) and transferred. After blockage, diluted antibodies (1 : 1000) (BAX, Ki67, Arg1, Tgf*β*1) were incubated with the membrane for 24 hours. After washing with PBS, secondary antibodies conjugated with horseradish peroxidase (HRP) were utilized and an enhanced chemiluminescence (ECL) substrate was added to visualize the protein bands. All chemicals were bought from Invitrogen, USA.

### 2.10. Transwell Assay and Wound Healing Experiment

CT26.WT cells were added to a transwell insert (Corning, 8 *μ*m diameter, USA) in the presence of PX serum for 24 hours. After the fixation by 4% paraformaldehyde (PFA), the cut insert membrane was subjected to DAPI (4′,6-diamidino-2-phenylindole) staining and examined by using the microscope.

10^5^CT26.WT cells were seeded in a 24-well plate at a density of 10^5^ at 37°C in a 5% CO_2_ incubator. When it reached 90% confluency, a scratch was made. After 24 hours, the migrated distance was measured. All chemicals were bought from Invitrogen, USA.

### 2.11. Enzyme-Linked Immunosorbent Assay (ELISA)

ELISA kits were bought from Abcam and utilized to examine the cytokine concentrations in the serum: interleukin (IL)-17 (sensitivity: 0.5 pg/ml, range: 6.25 pg/ml–400 pg/ml), IL-6 (sensitivity: 11.3 pg/ml, range: 15.6 pg/ml–1000 pg/ml), interferon*γ* (IFN-*γ*) (sensitivity: 1.3 pg/ml, range: 1.3 pg/ml–2000 pg/ml), and tumor necrosis factor*α* (TNF*α*) (sensitivity: 9.1 pg/ml, range: 46.88 pg/ml–3000 pg/ml).

### 2.12. Statistical Analysis

Data are means ± SEM. An unpaired Student's *t*-test was used to compare the difference between the two groups. A Cox proportional hazard model was established to indicate PX targets associated with the survival probability in COAD patients. A hazard ratio (HR) with 95% confidence intervals and a log-rank *P* value were calculated via univariate survival analysis, and a *P* value of <0.05 was considered statistically significant.

## 3. Results

### 3.1. HIT Network of PX and XH

**Figure 1:**
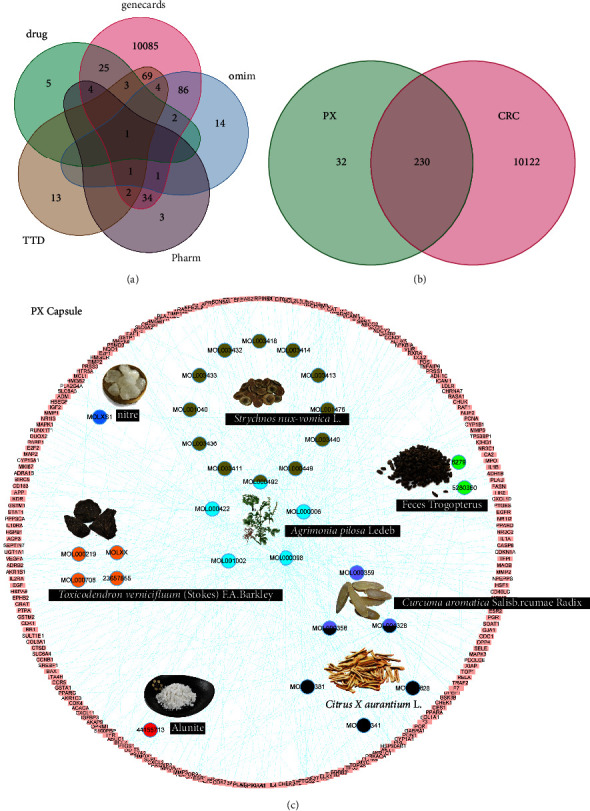
The herb-ingredient-target network of PX. Venn analysis showing colorectal cancer (CRC)-related genes from five databases: DrugBank, GeneCard, OMIN, PharmGkb, and TTD (a) and the shared targets between PX and CRC (b), network of herbs and compounds as well as all the potential targets (c).

The PX ingredients had 171 active compounds (Supplementary Table 1) and shared 230 putative targets with a total of 10352-CRC genes (Figures 1(a) and 1(b)) (Supplementary Table 2). A HIT network representing the interactions between the active components of the PX formula and targets was established: pink rectangle nodes were the shared genes, and nodes inside represented active components (Figure 1(c)). As illustrated in the pie chart of target proportion, AL occupied 43% of the formula (Supplementary Figure 2A). KEGG analysis predicted the enriched pathways involved in the formula (Supplementary Figure 2B) and each herb (Supplementary Figure 2C) combating CRC.

The HIT network of XH was also established. Musk, *Commiphora myrrha* (Nees) Engl., Bovis calculus, and *Boswellia sacra* Flueck., shared 179 genes with CRC, and Myrrha was responsible for 78% of the targets (Supplementary Figure 6). KEGG analysis predicted the enriched pathways (Supplementary Figure 7).

### 3.2. PX-Targeted Genes Correlate with the Survival Probability in CRC Patients

**Figure 2:**
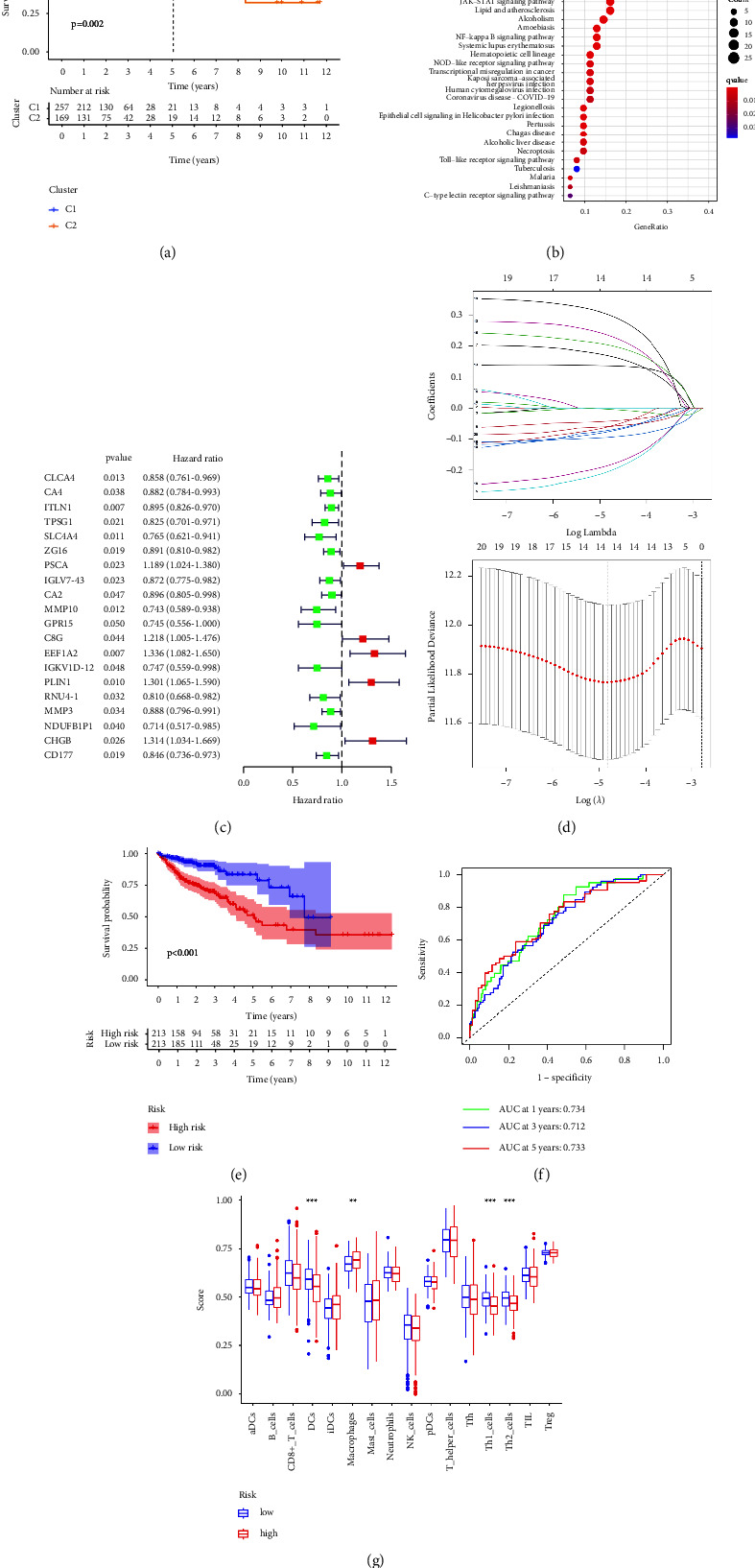
The therapeutic value of the PX formula in CRC. Kaplan–Meier curves demonstrating the prognostic value of clusters in patients with COAD (a), KEGG analysis showing the enriched pathways of those PX targets with prognostic value (b), uni-Cox analysis showing the hazard ratio of PX targets (c), lasso model establishment (d), Kaplan–Meier curves indicating the correlation of survival with high-risk and low-risk groups (e), ROC curve demonstrating the sensitivity and specificity of the lasso model (f), The correlation of immune cell infiltrates with PX targets (g), ^*∗*^*p* < 0.05,  ^*∗∗*^*p* < 0.01,  ^*∗∗∗*^*p* < 0.001 indicates a statistically significant difference from the saline group.

Utilizing consensus clustering, a total of 426 patients with COAD were divided into two clusters, which showed a correlation with the survival probability (Figure 2(a)). The DEGs between the two clusters were enriched in inflammatory reactions, pathogen-recognition pathways including Toll-like receptor and NOD-like receptor signaling pathways, and infectious diseases (Figure 2(b)). Univariate Cox regression analysis yielded 20 PX-targeted genes associated with the survival in COAD patients (Figure 2(c)), and lasso regression analysis selected 14 genes as prognostic signatures (ITLN1, TPSG1, SLC4A4, PSCA, IGLV7-43, MMP10, C8G, EEF1A2, IGKV1D-12, PLIN1, RNU4-1, NDUFB1P1, CHGB, and CD177) (Figure 2(d)). The high-risk group exhibited a lower survival probability than the low-risk group (Figure 2(e)), and the AUC values were 0.734, 0.712, and 0.733, respectively (Figure 2(f)), indicating a favorable discrimination performance for outcome prediction. ssGSEA demonstrated that these signatures were closely related to the effector functions of M*φ*s, dendritic cells, and Th1 and Th2 cells (Figure 2(g)).

### 3.3. The Antitumor Effect of the PX Formula on CRC Murine Models

**Figure 3:**
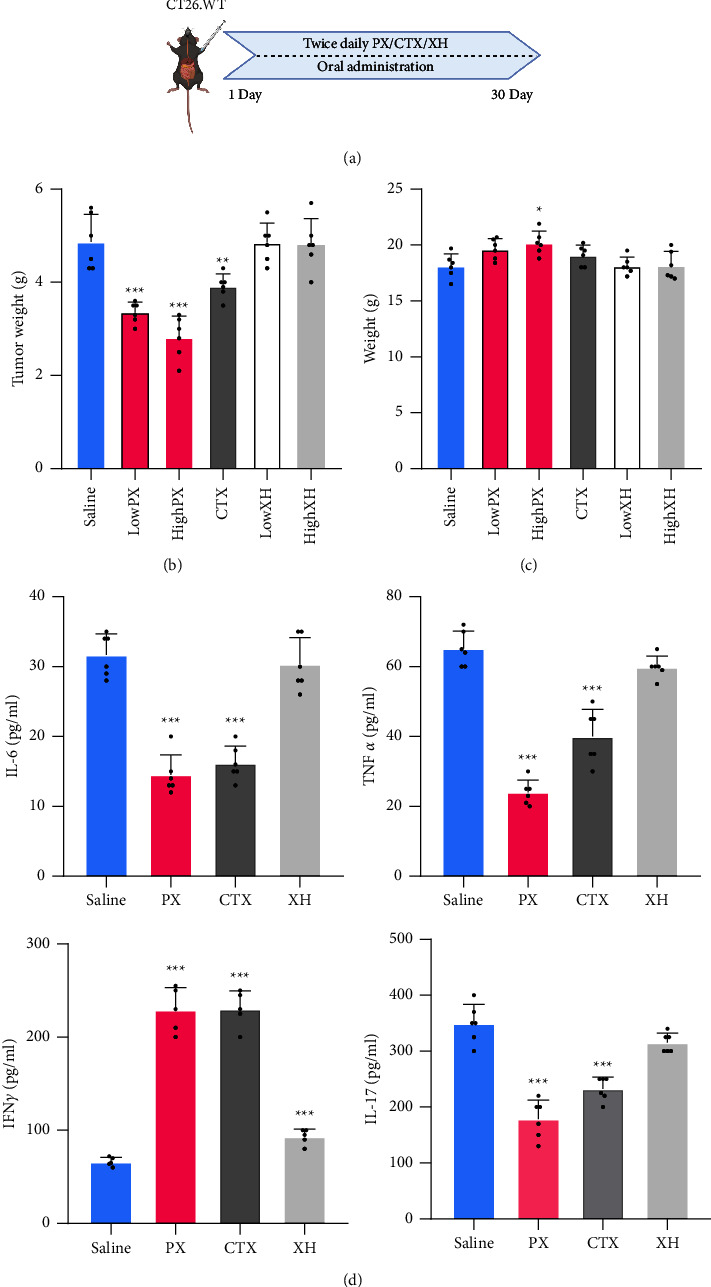
Pingxiao formula suppresses colorectal cancer progression. The flow chart of experimental design (a), tumor weight (b), mice weight (c), and IL-6, IL-17, IFN*γ*, and TNF*α* levels (d) from colorectal cancer murine models after PX treatment. ^*∗*^*p* < 0.05,  ^*∗∗*^*p* < 0.01 and ^*∗∗∗*^*p* < 0.001 indicate a statistically significant difference from saline group.

LC-MS/MS confirmed PX components (Supplementary Figure 1) (Supplementary Table 3). The schematic of the experimental design is shown (Figure 3(a)). As illustrated, the PX formula suppressed the tumor volume at both concentrations and exhibited a stronger tumor-suppressing capacity than cyclophosphamide (CTX) and XH (Figures 3(b) and 3(c)). Moreover, we observed that the high dose of PX mitigated the weight loss of CRC mice (Figure 3(d)). The high dose of PX was selected for further experiments. Moreover, the high dose of PX or CTX significantly hampered the concentrations of IL-6, IL-17, and TNF*α*, whereas it increased IFN*γ* secretion, whereas in this case, XH failed (Figure 3(d)). Altogether, PX is more potential than the XH formula.

### 3.4. PX Formula Promotes Gut Immunity and Induces Colorectal Cancer Cell Apoptosis

**Figure 4:**
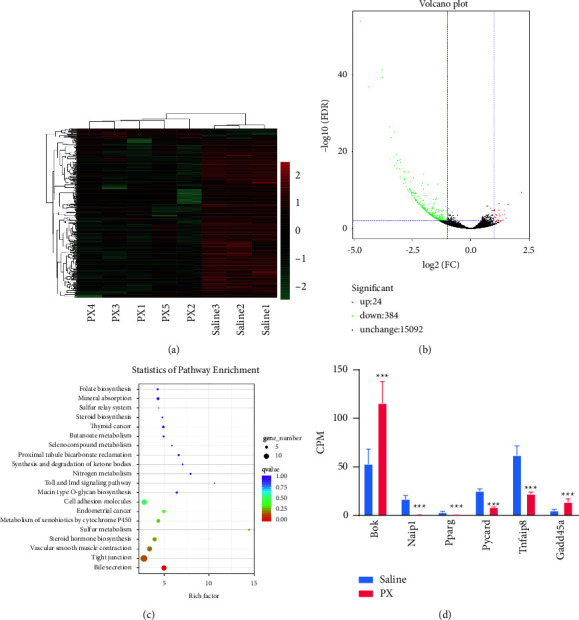
Gene expression profiles in PX-treated tumors. The heatmap (a) and volcano plot (b) showing the differentially expressed genes (DEGs) between saline and PX groups; KEGG analysis of DEGs (c), counts per million analysis of the expression of key genes (d). ^*∗∗∗*^*p* < 0.001 indicates a statistically significant difference between the groups.

Solid tumor tissue was collected from CRC mice with or without PX treatment. Saline and PX groups clustered distinctly from each other (Figure 4(a)). Among the differentially expressed genes (DEGs) (Figure 4(b)), there were 24 up-regulated genes and 384 down-regulated genes after PX administration, which were enriched in multiple cancers, cell adhesion molecules, the Toll and Imd signaling pathways, and cytochrome P450. Specifically, the apoptosis-related gene BAX was significantly increased after PX treatment, while Naip1, Pparg, PYCARD, Tnfaip8, and Gadd45a were decreased (Figure 4(d)).

### 3.5. Fecal Microbiota and Metabolite Analysis

**Figure 5:**
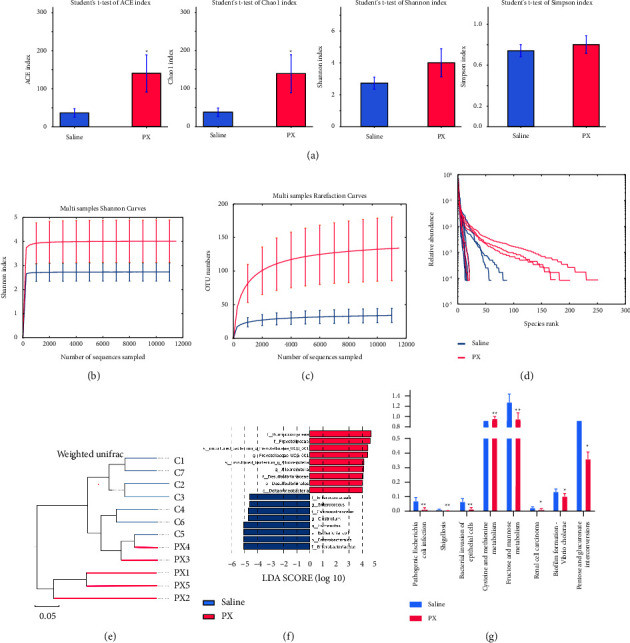
Profiles of microbial communities with PX treatment. Alpha diversity of microbial communities in mice undergoing CRC and treated with PX (a), Shannon curves (b), rarefaction analysis (c), rank abundance curve (d), and PERMANOVA (e) illustrating the clustering of gut flora between saline and PX-treated groups; LEFSe (f) illustrating the abundance of bacterial species.

Given the predicted antibacterial effects of PX and XH by network pharmacology analysis, we explored alterations in the gut microbiota. All sample libraries covered >99%, suggesting a sufficient library size to represent most microbes (Supplementary Table 4). Shannon curves with flat ends and rarefaction analysis showed that the OTUs' numbers of both groups adequately presented most species and reached saturation (Figures 5(b) and 5(c)). PX treatment significantly enhanced Ace and Chao indices and also increased Shannon as well as Simpson, albeit without statistical significance (Figure 5(a)). The rank abundance curve ranking the OTU abundance of each sample by size represents both the species richness and species evenness in samples. The wider the curve is, the more abundant the composition of species is; the flatter the shape is, the more even the composition of species is (Figure 5(d)). Weighted unifracdistance with PERMANOVA showed that PX treatment significantly altered the *β*-diversity of commensal microorganisms (Figure 5(e)). Linear discriminant analysis coupled with effect size measures (LEFSe) showed that PX treatment suppressed the abundance of *Clostridium*, *Escherichia*, and *Enterococcus*, while it increased Prevotellaceae*UCG 001* and *Alloprevotella* (Figure 5(f)). Tax4Fun [18] analysis predicted that PX decreased pathogenic bacterial infection and the metabolism of fructose, mannose, cysteine, and methionine (Figure 5(g)).

Compared with PX, XH failed to influence the alpha diversity of the microorganisms in CRC murine models (Supplementary Figure 4A) (Supplementary Table 5). PCoA and a heatmap based on Bray Curtis suggested that XH treatment did not change the beta diversity of microbiota (Supplementary Figures 4B and 4C). Krona demonstrated that the abundance of Clostridia increased from 21% to 26% (Supplementary Figure 4D).

### 3.6. PX Formula Stimulates the Classical Activation of M*φ*s

**Figure 6:**
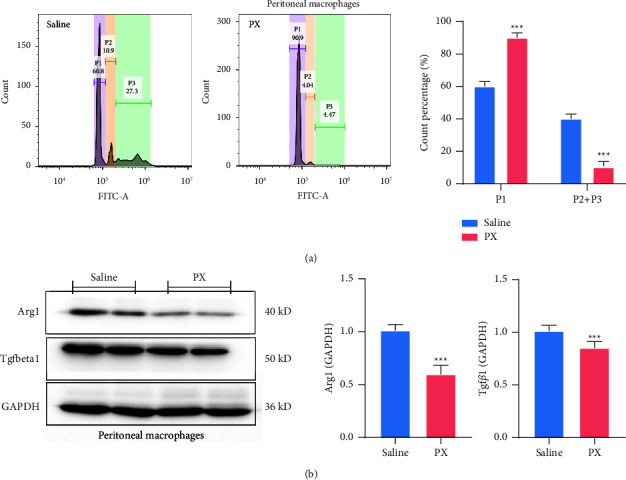
PX formula facilitates the classical activation of macrophages. Bar charts (*n* = 4) presenting the phagocytic capacity of peritoneal macrophages in CRC murine models after PX treatment, one microparticle engulfment (P1), two microparticles (P2), and more than two microparticles (P3) (a). Original western blotting demonstrating the protein levels of Arg1 and Tgf*β*1 in peritoneal macrophages (b). ^*∗∗∗*^*p* < 0.001 indicates a statistically significant difference from the saline group.

Given the pivotal role of TME in carcinogenesis and the predicted involvement of M*φ*s by the WGCNA, peritoneal M*φ*s were collected from CRC murine models. PX treatment suppressed the proportion of P2-3 peritoneal M*φ*s from 38.2% to 8.51% (Figure 6(a)). The protein abundance of Arg1 and Tgf*β*1, as M2 M*φ* markers, was significantly decreased in peritoneal M*φ*s after PX treatment (Figure 6(b)), indicating the inhibition of M2 M*φ* transition.

### 3.7. PX Serum Suppresses CW26.WT Cell Migration and Invasion

**Figure 7:**
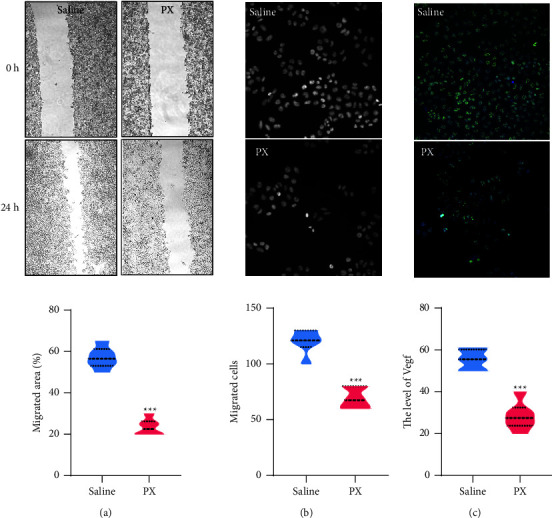
PX formula inhibits colorectal cancer cell migration. The wound healing assay (a) and transwell experiment (b) showing the migration of CT26.WT cells in the presence of PX serum; immunofluorescent staining showing the VEGF expression (c) in CT26.WT cells after the treatment of PX serum for 24 hours. ^*∗∗∗*^*p* < 0.001 indicates a statistically significant difference from the saline group.

To examine the direct antineoplastic role of the PX formula, the serum from CRC murine models after PX treatment was added to CW26.WT cells. An MTT assay showed that PX serum treatment did not affect the viability of CT26.WT cells (Supplementary Figure 5). PX serum significantly suppressed the migration capacity of cancer cells after 24-hour incubation, as evidenced by wound healing assays and transwell experiments (Figures 7(a) and 7(b)), and markedly inhibited the expression of VEGF (Figure 7(c)).

### 3.8. PX Formula Reshapes TME and Induces Cancer Cell Apoptosis

**Figure 8:**
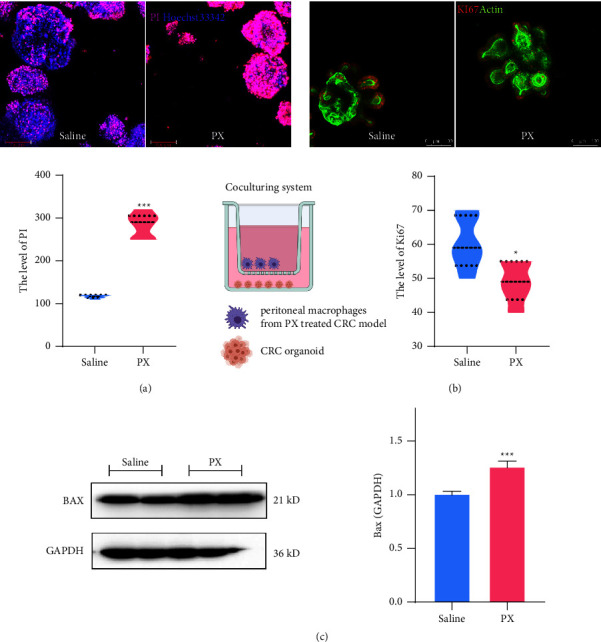
PX-treated peritoneal macrophages suppress tumor-derived organoid proliferation. Immunofluorescent staining showing the hoechst33342/PI (a), Ki67 expression (b) in tumor-derived organoids with the coculturing of PX-treated peritoneal macrophages for 24 hours. Original western blotting demonstrating the protein levels of BAX in peritoneal macrophages (c). ^*∗*^*p* < 0.05,  ^*∗∗*^*p* < 0.01 and ^*∗∗∗*^*p* < 0.001 indicate a statistically significant difference from the saline group, and # indicates statistically significant difference from the UC group.

Colorectal tumor-derived organoids (cTDOs) were cultured as a model to examine the proliferation capacity of cancer stem cells, which recapitulates the neoplastic progression within TME. An *ex-vivo* CRC cellular model was established by coculturing cTDOs with peritoneal M*φ*s from PX-treated CRC murine models. As illustrated, PX-treated peritoneal M*φ*s induced cTDO apoptosis, as evidenced by PI staining and elevated Bax protein abundance (Figures 8(a) and 8(c)). Moreover, the expression of Ki67 in cTDOs was significantly hindered by coculturing with PX-treated M*φ*s (Figure 8(b)).

## 4. Discussion

In the present study, we provide evidence of the anticancer efficacy of the PX formula against CRC and demonstrate the underlying mechanisms. As the third most common cancer worldwide, CRC develops in the setting of hereditary cancer syndromes or from inflammatory bowel disease (IBD) [19], and its complex etiology involves a broad spectrum of causal factors, including environment, genetic susceptibility, and diet [4]. Whilst the mortality rate of CRC patients has been decreased due to effective cancer screening measures and multiple anticancer therapies, those patients who are not suitable for surgery or chemotherapy, or with cancer cachexia, are overlooked, and finding a potential drug that is low-costing and effective yet of low toxicity is urgent. Accumulated investigations have demonstrated a strong regulation of cancer progression and metastasis by the reciprocal interactions between cell-intrinsic and cell-extrinsic mechanisms within the TME [20, 21]. In this context, targeting TME portends an ideal choice either as a monotherapy or an adjuvant in combination with other agents as well as chemotherapy. TCM has multiple components fulfilling synergistic functions with minimized side effects based on Shang Han Lun, which fits with the concept of targeting TME.

PX and XH are widely used in China to treat cancers [8]. In the present study, we inoculated cancer cells into the mice and administered these prophylaxis remedies simultaneously and compared their antitumor effects on day 30. Our *in-vivo* experiments demonstrated that the PX formula exhibited a stronger antitumor effect than XH treatment, as evidenced by the suppressed tumor size and inhibited levels of IL-17, IL-6, and TNF*α*, which are the best characterized independent risk factors for CRC [22–24] and increased IFN*γ* expression that inhibits the expansion of disseminated colorectal cancer cells [25]. KEGG analysis showed that PX-target genes were enriched in inflammatory reactions, microbe infections, drug resistance, and carcinogenesis, including CRC and bladder cancer, which was consistent with the RNAseq results of murine CRC models after PX treatment. In short, the PX formula presents a promising drug that is able to orchestrate the complex interactions within the TME, for instance, the communications between cancer cells and immune cell populations, microbes, and antigen-presenting cells.

Given the high complexity of CRC pathogenesis, we delineated the pharmacological logic of the PX formula from seven aspects:

1. Reestablishing the immune status within the tumor: in developed cancers, immune surveillance is mostly missing and the immune-suppressive TME is established. TME is critical for carcinogenesis and cancer maintenance by supporting tumor growth, invasion, metastasis, and hiding from T-cell detection and traditional anticancer therapy. However, not all the immune cells within the TME represent an attempt to impair tumor immune surveillance and enable tumor cells to proliferate rapidly. The combination of AF, CR, AL, and TV tends to induce immunity and eradicate neoplastic cells by shaping the plasticity potentials of M*φ*s and Th17 cells, both of which are protumorigenic in CRC [26–28], as evidenced by the enriched IL-17 and TNF signaling pathways. M*φ*s are immune cells with high plasticity and can transit to different phenotypes in response to diverse stimuli. The classically activated (M1) M*φ*s are proinflammatory and induce antitumor immunity, while the alternatively activated (M2) M*φ*s contribute to wound healing and facilitate cancer cells escaping from the host immune reactions [21, 29]. Tumor-infiltrating M*φ*s (TAMs) mostly take on an M2-like phenotype and contribute to immune escape, allowing the transformation of neoplastic cells. Herewith, changing M2 M*φ*s toward M1 M*φ*s is one of the prospective aims of cancer immunotherapy [30]. Based on TCGA database, we found a close association of PX targets with the survival probability in patients with CRC, as well as with TAM infiltrating, which corroborates the therapeutic value of PX in the clinic. Additionally, our in vitro experiments corroborated the induction of proinflammatory polarization of M*φ*s, which in turn instigated the apoptosis of cancer stem cells and inhibited neoplastic cell migration. Collectively, the PX formula is able to reverse the tumor-promoting phenotype of TAMs and thus reshape the communication between TAMs and other cell populations or microbes, which has also been considered a causal factor for neoplastic processes and influences the responsiveness to cancer therapeutics [31].
2. Induction of metastatic cancer cells apoptosis: in our in vitro experiments, we observed that the peritoneal M*φ*s from the PX mice suppressed the migration capacity of cancer cells shown in the transwell experiments and wound healing assays and inhibited the expression of VEGF in cancer cells, indicating decreased metastatic dissemination [32]. Moreover, the peritoneal M*φ*s from the PX mice induced apoptosis as evidenced by the increased PI level and BAX protein abundance and reduced proliferation-related Ki67 expression [33] of cTDOs, which are cancer stem cells responsible for early neoplastic progression and expansion of disseminated cancer cells [34]. Altogether, the PX therapy could inhibit metastatic cancer cell migration and proliferation, and promote apoptosis, which might be accomplished by AF, CR, AL, and TV [35, 36].
3. Altering the pathological microbiota communities: perturbations of the colonic microorganism communities are conducive to the carcinogenesis and metastasis of CRC, which exacerbate the colonization of pathogenic taxa at the expense of probiotics and hence produce cancer-promoting metabolites [37]. PX treatment successfully rescued the decrease in microbiota richness in murine models of CRC and significantly suppressed the colonization of the *genus Clostridium, Escherichia*, and *Enterococcus*, which are considered human pathogens and associated with colorectal carcinogenesis by inducing colonic inflammation and stimulating neoplastic signaling pathways [38–40]. Moreover, PX markedly increased the levels of probiotic gut microbiota [41] such as the *genus Alloprevotella* and *Prevotellaceae* UCG 001. Based on KEGG analysis and RNA sequencing, PX exhibits a strong microbicidal effect by using AF, CR, AL, and TV, as well as white alunite, which is a natural product with a potential antivirus [42] and antibacterial capacity [43]. Compared with the 16S rRNA sequencing results of the PX formula, no significant alteration in microbial communities has been observed in the XH-treated CRC group, which might explain the poor efficacy of the XH formula.
4. Reversing drug resistance: cancer-related mortality is mainly attributed to the development of drug resistance. Platinum-based drugs and epidermal growth factor receptor (EGFR) inhibitors have been used widely against human tumors [44, 45], and endocrine therapy has been considered the major treatment of estrogen receptor (ER)-positive breast cancer [46]. Based on the system pharmacology analysis, the PX formula influenced EGFR tyrosine kinase inhibitors, platinum drugs, and endocrine resistance, by the combination of CL, CR, AL, and TV.
5. Reprogramming of amino acid metabolism and nervous and humoral regulation: to suffice the biosynthetic demands of unlimited proliferated cancer cells from nutrient-poor TME, an abundant supply of amino acids is pivotal [47]. As a major component of the PX formula, SS was dedicated to the reprogramming of deregulated amino acid metabolism, including tyrosine, tryptophan, histidine, glycine, serine, and threonine, and was associated with serotonergic synapse activities, neuroactive ligand-receptor interaction, and neurotransmitter metabolic process, which orchestrates gut homeostasis and relieves mental stress [48–50]. Moreover, AF and CR modulate the thyroid hormone signaling pathway, which is crucial for aging and age-related diseases, including cancer [51]. In addition, the enriched estrogen signaling pathway also indicates that PX would also exert an effective anticancer capacity against breast cancer.
6. Malignancy forms a thrombophilic state by increasing venous stasis, which portends a poor prognosis [52]. FT promotes blood circulation and relieves blood stasis [53] and is also a source of dietary lignans with antiestrogenic, antiangiogenic, proapoptotic, and antioxidant mechanisms [54]. Despite the concerns regarding the risk of infection by zoonotic agents [55], the strong antibacterial combination of AF, CR, AL, and TV, as well as alunite, would decrease the possibility of FT-caused infection.
7. Last but not least, the multiple components might raise a concern about the absorption and bioavailability of the PX formula. Lines of evidence have demonstrated that mirabilite could alter the pharmacokinetic behaviors of herb components by orchestrating the absorption of each compound [56]. Consistently, RNA sequencing corroborated that the PX formula modulated the cytochrome P450 system, the key enzyme responsible for drug metabolism [57].

Collectively, these herbs in the PX formula work synergistically to instigate the host anticancer immunity and thus stimulate cancer cell apoptosis, suppress infections, and reshape microbiota communities, establishing a tumor-eradicating TME, and restore the homeostasis of humoral. Although the 179 XH targets are also enriched in various cancers, inflammation, apoptotic signaling, nervous and humoral regulation, and estrogen signaling pathways, the shortcomings of the XH formula might be attributed to the neglect of drug metabolism and resistance, humoral circulation, energy consumption, and microbiota composition. In short, the PX formula exerts a more successful therapeutic effect than XH and might be administered either as an adjuvant in combination with other therapeutic strategies or as a monotherapy for those patients who could not suffer surgery as well as chemotherapy. Especially, the combined utilization of the PX formula with other treatment modalities in traditional Chinese medicine such as acupuncture [58, 59], which shows potent efficacy in alleviating fatigue and cancer pain, might considerably improve the quality of life of patients with CRC. Assuming that our murine CRC models established by orthotopic cecal injection of CT26.WT cells lack genetic diversity and are of modest value for predicting clinical relevance [60–62], WGCNA based on TCGA database was conducted and corroborated the therapeutic value.

## 5. Conclusion

We posit that the antineoplastic mechanism of PX involves the induction of anticancer immunity, the establishment of healthy microbiota composition, and the improvement of drug metabolism.

## Figures and Tables

**Table 1:** Components in the Pingxiao and Xihuang formula.

	Chinese name	Material
*Scientific names of the herb in PX*		
Alunite	Bai Fan	Crystal
*Toxicodendron vernicifluum* (Stokes) F. A. Barkley (Anacardiaceae)	Gan Qi	Pitch
*Strychnos nux-vomica* L. (Loganiaceae)	Ma Qian Zi	Seed
Nitre	Xiao Shi	Crystal
*Agrimonia pilosa* Ledeb.(Rosaceae)	Xian He Cao	Rootstalk
*Curcuma aromatica* Salisb.(Zingiberaceae)	Yu Jin	Rootstalk
*Citrus* × *aurantium* L. (Rutaceae)	Zhi Ke	Pulp
*Feces trogopterus*	Wu Ling Zhi	Feces

*Scientific names of the herb in XH*		
Musk	She Xiang	Moschus
*Commiphora myrrha* (Nees) Engl. (Burseraceae)	Mo Yao	Pitch
Bovis calculus	Niu Huang	Cow bezoar
*Boswellia sacra* Flueck. (Burseraceae)	Ru Xiang	Pitch

## Data Availability

The data used to support the findings of this study are available upon request.

## Abbreviations

CC: Colorectal cancer
IOs: Intestinal organoids
M*φ*s: Macrophages
PX: Pingxiao
XH: Xihuang
TCM: Traditional Chinese medicine
TME: Tumor microenvironment
BC: Betweenness centrality
DC: Degree centrality
EC: Eigenvector centrality
LAC: Local average connectivity
NC: Network centrality
GO: Gene ontology
KEGG: Kyoto encyclopedia of genes and genomes
AUC: Area under the curve
VEGF: Vascular Endothelial growth factor.

## Supplementary Materials

Supplementary Materials
*Supplementary Table 1.* Active components of PX. *Supplementary Table 2.*CRC-related genes in databases and intersected genes. *Supplementary Table 3.* LC-MS/MS analysis of the PX formula. *Supplementary Table 4.* Alpha diversity indices of the gut microbiota after PX treatment. *Supplementary Table 5.* Alpha diversity indices of the gut microbiota after XH treatment. *Supplementary Figure 1.* LC-MS/MS identified PX components. *Supplementary Figure 2.* The therapeutic effect of each herb in CRC. The pie chart illustrating the target genes of each herb in CRC (A); GO and KEGG analysis showing the enrichment of herb-target genes (B), target genes of each herb (C). *Supplementary Figure 3.* The therapeutic effect of PX and XH on the progression of CRC. *Supplementary Figure 4.* Alpha diversity of microbial communities in CRC mice after XH treatment. *Supplementary Figure 5.* The MTT assay evaluating the toxicity of the PX serum. *Supplementary Figure 6.* The herb-ingredient-target network of XH. Shared targets between XH and CRC (A); a pie chart showing the target genes of each herb in CRC (B); a network of herbs and compounds as well as all the potential targets (C). *Supplementary Figure 7.* The therapeutic effect of each herb in XH.
