# Supplementary material for: Pharmacological Mechanism of Pingxiao Formula against Colorectal Cancer
Source: Evid Based Complement Alternat Med. 2022 Dec 20;2022:7884740. doi: 10.1155/2022/7884740 (PMC9794442; doi:10.1155/2022/7884740)
Supplement: Supplementary Materials — Supplementary Table 1. Active components of PX. Supplementary Table 2.CRC-related genes in databases and intersected genes. Supplementary Table 3. LC-MS/MS analysis of the PX formula. Supplementary Table 4. Alpha diversity indices of the gut microbiota after PX treatment. Supplementary Table 5. Alpha diversity indices of the gut microbiota after XH treatment. Supplementary Figure 1. LC-MS/MS identified PX components. Supplementary Figure 2. The therapeutic effect of each herb in CRC. The pie chart illustrating the target genes of each herb in CRC (A); GO and KEGG analysis showing the enrichment of herb-target genes (B), target genes of each herb (C). Supplementary Figure 3. The therapeutic effect of PX and XH on the progression of CRC. Supplementary Figure 4. Alpha diversity of microbial communities in CRC mice after XH treatment. Supplementary Figure 5. The MTT assay evaluating the toxicity of the PX serum. Supplementary Figure 6. The herb-ingredient-target network of XH. Shared targets between XH and CRC (A); a pie chart showing the target genes of each herb in CRC (B); a network of herbs and compounds as well as all the potential targets (C). Supplementary Figure 7. The therapeutic effect of each herb in XH. [file 7884740.f1.zip › Supplementary figure legends.docx]

**Supplementary Table 1. Active components of PX**

**Supplementary Table 2. CRC-related genes in databases and intersected genes**

**Supplementary Table 3. LC-MS/MS analysis of the PX formula**

**Supplementary Table 4. Alpha diversity indices of the gut microbiota after PX treatment**

**Supplementary Table 5. Alpha diversity indices of the gut microbiota after XH treatment**

**Supplementary Figure 1. LC-MS/MS identified PX components**

**Supplementary Figure 2. The therapeutic effect of each herb in CRC**

Pie chart illustrating the target genes of each herb in CRC (A); GO and KEGG analysis showing the enrichment of herb-target genes (B), target genes of each herb (C).

**Supplementary Figure 3. The therapeutic effect of PX and XH on the progression of CRC**

**Supplementary Figure 4. Alpha-diversity of microbial communities in CRC mice after XH treatment.**

**Supplementary Figure 5. MTT assay evaluating the toxicity of the PX serum**

**Supplementary Figure 6. The Herb-Ingredient-Target network of XH**

Shared targets between XH and CRC (A); Pie chart showing the target genes of each herb in CRC (B); Network of herbs and compounds as well as all the potential targets (C).

**Supplementary Figure 7. The therapeutic effect of each herb in XH**
